# The regulation of cell wall lignification and lignin biosynthesis during pigmentation of winter jujube

**DOI:** 10.1038/s41438-021-00670-4

**Published:** 2021-11-01

**Authors:** Qiong Zhang, Lihu Wang, Zhongtang Wang, Rentang Zhang, Ping Liu, Mengjun Liu, Zhiguo Liu, Zhihui Zhao, Lili Wang, Xin Chen, Haifeng Xu

**Affiliations:** 1 Shandong Institute of Pomology, Tai’an, Shandong 271000 China; 2 School of Landscape and Ecological Engineering, Hebei University of Engineering, Handan, Hebei 056038 China; 3 College of Food Science and Engineering, Shandong Agricultural University, 61 Daizong Street, Tai’an, 271018 Shandong P.R. China; 4 Research Center of Chinese Jujube, Hebei Agricultural University, Baoding, Hebei 071000 China

**Keywords:** Secondary metabolism, Plant molecular biology

## Abstract

Fruit lignification is due to lignin deposition in the cell wall during cell development. However, there are few studies on the regulation of cell wall lignification and lignin biosynthesis during fruit pigmentation. In this study, we investigated the regulation of cell wall lignification and lignin biosynthesis during pigmentation of winter jujube. The cellulose content decreased, while the lignin content increased in the winter jujube pericarp during pigmentation. Safranin O-fast green staining showed that the cellulose content was higher in the cell wall of winter jujube prior to pigmentation, whereas the lignin in the cell wall increased after pigmentation. The thickness of the epidermal cells decreased with pericarp pigmentation. A combined metabolomics and transcriptomics analysis showed that guaiacyl-syringyl (G-S) lignin was the main lignin type in the pericarp of winter jujube, and *F5H* (*LOC107424406*) and *CCR* (*LOC107420974*) were preliminarily identified as the key genes modulating lignin biosynthesis in winter jujube. Seventeen MYB and six NAC transcription factors (TFs) with potential regulation of lignin biosynthesis were screened out based on phylogenetic analysis. Three MYB and two NAC TFs were selected as candidate genes and further studied in detail. *Arabidopsis* ectopic expression and winter jujube pericarp injection of the candidate genes indicated that the MYB activator (*LOC107425254*) and the MYB repressor (*LOC107415078*) control lignin biosynthesis by regulating *CCR* and *F5H*, while the NAC (*LOC107435239*) TF promotes *F5H* expression and positively regulates lignin biosynthesis. These findings revealed the lignin biosynthetic pathway and associated genes during pigmentation of winter jujube pericarp and provide a basis for further research on lignin regulation.

## Introduction

Jujube (*Ziziphus jujuba* Mill.) is a medicinal and edible fruit that originates from China. Owing to various characteristics, such as tolerance of barren environments, salt-alkali conditions, drought, wind and sand, and early and quick harvesting, jujube trees are pioneers in promoting economic development and ecological construction in sand-alkali-arid regions^1^. Jujube fruit is nutritious and rich in a variety of functionally active components. Recent studies have found that ursolic acid and oleanolic acid in jujube fruit extracts inhibit breast cancer metastasis and cell apoptosis^2^. Moreover, polyphenols in the jujube pericarp were reported to effectively protect against myocardial ischemia caused by isoproterenol^3^.

Lignin is an important component of cell walls that maintains the rigidity of cell walls and provides mechanical support for the cells. Its hydrophobicity can prevent water loss. The secondary lignification of cell walls plays a key role in the growth and development of plants, as well as in the resistance to various biotic and abiotic stresses^4^. Lignin contains three monomers, namely, *p*-hydroxyphenyl lignin (H lignin), syringyl lignin (S lignin), and guaiacyl lignin (G lignin). Lignin biosynthesis is one of the most important flavonoid biosynthesis pathways, while the *PAL*, *C4H*, and *4CL* genes serve as the common structural genes of the flavonoid and lignin biosynthesis pathways. Silencing of the *PAL* gene in tobacco resulted in decreased flavonoid and lignin contents and impaired plant growth and development^5^. Knockout of the *C4H* gene in Arabidopsis (*Arabidopsis thaliana* Heynh.) blocked the biosynthesis of flavonoid and lignin^6^. In addition, downregulating the expression of various genes, including cinnamoyl CoA reductase (*CCR*), cinnamyl alcohol dehydrogenase (*CAD*), and hydroxycinnamoyl-CoA *(HCT*), in Arabidopsis reduced the lignin content but increased the flavonoid content, and the plants were smaller with stunted development^7,8^.

Lignin biosynthesis is regulated by MYB TFs. Some MYB TFs can simultaneously regulate flavonoid and lignin synthesis in positive or negative directions. For instance, *CmMYB8* in chrysanthemum (*Dendranthema morifolium* Tzvel.) simultaneously represses the accumulation of lignin and flavonoids^9^. However, most MYB TFs can only positively regulate flavonoid or lignin synthesis. For example, the overexpression of tomato *SlMYB4* significantly reduced the expression levels of structural genes such as *PAL, 4CL, C4H*, and *CCR* and decreased lignin accumulation. In contrast, there was no significant change in the flavonoid content since *SlMYB4* could not bind to the *SlCHS1* promoter^10^. The overexpression of grape *VvMYB5a* and *VvMYB5b* in petunia upregulated the expression of the *PAL*, *C4H*, and *4CL* genes, increased the content of anthocyanin, downregulated *CCoAOMT1* expression, and reduced lignin content^11^. Furthermore, NAC is another specific TF in lignin biosynthesis. It has been reported that the fruit lignification of pear (*Pyrus pyrifolia*) is regulated by *PpNAC187*. Specifically, the overexpression of *PpNAC187* upregulated the expression of the *CCR* and *COMT* genes and increased the content of lignin^12^. Another study showed that the overexpression of *PdWND3A* in poplar (*Populus euphratica* Oliv.) upregulated the expression of the *F5H1* gene and promoted the biosynthesis of lignin^13^.

The pigmentation process of the jujube pericarp occurs with the ripening of the fruit and is accompanied by the deposition of insoluble fiber in the cell walls. However, studies on the regulation of cell wall lignification and lignin biosynthesis in jujube are lacking. Herein, we analyzed the changes in insoluble fiber and pericarp cells and the regulation of lignin biosynthesis during pigmentation of winter jujube (*Z. jujuba* Mill. cv. Dongzao) using biochemical, metabolomic, transcriptomic, and transgenic approaches.

## Results

### Changes in insoluble fiber contents and cell structures during the pigmentation process of winter jujube pericarp

**Fig. 1:**
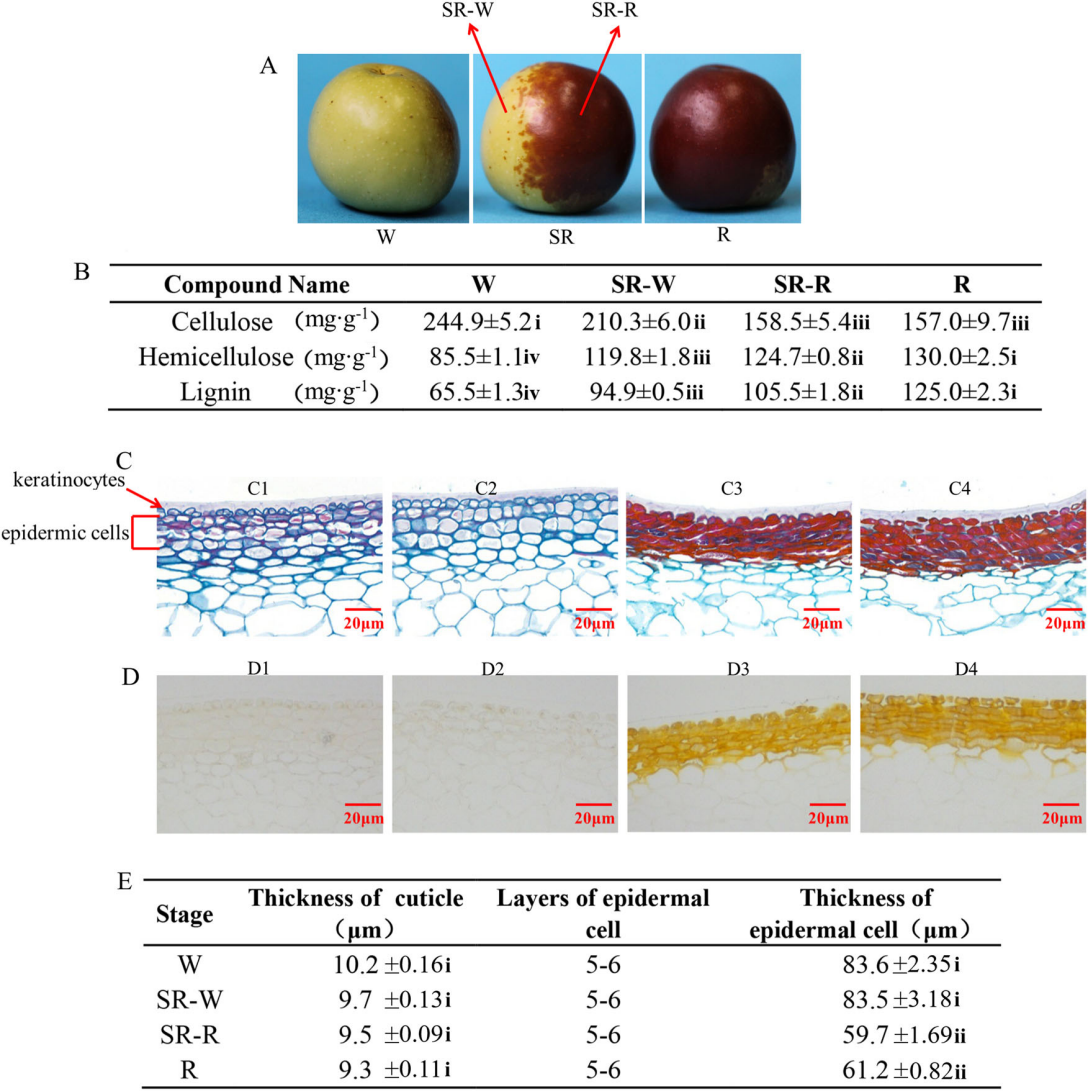
Insoluble fiber contents and dynamic changes of pericarp cells during the pigmentation of winter jujube. **A** Phenotypes of winter jujube in different coloring periods. **B** The contents of lignin, cellulose, and hemicellulose during the pigmentation of winter jujube. **C** Paraffin sections of pericarp using safranin O-fast green staining in different coloring periods. C1: pericarp of W, C2: white pericarp of SR, C3: red pericarp of SR, C4: pericarp of R. **D** Paraffin sections of pericarp in different coloring periods. D1: pericarp of W, D2: white pericarp of SR, D3: red pericarp of SR, D4: pericarp of R. **E** Layers and thickness of pericarp cells in different coloring periods. W: white stage; SR-W: white pericarp of the semi-red stage; SR-R: red pericarp of the semi-red stage; R: full-red stage

The pericarp color of winter jujube fruit at different periods of maturation varies from white (W) to semi-red (SR) to full-red (R) (Fig. 1A). As shown in Fig. 1B, cellulose was the main component in the pericarp of winter jujube, followed by hemicellulose and lignin. The cellulose content gradually decreased from the W stage to the R stage. In contrast, the hemicellulose and lignin contents of the winter jujube pericarp were the lowest in the W stage and increased by 52 and 91% in the R stage, respectively.

The epidermal cells of jujube were arranged closely and neatly at the W stage and SR-W stage, with oblate or oblong shapes (Fig. 1C). At the SR-R and R stages, the epidermal cells of winter jujube were significantly shriveled with increased transverse diameters and decreased longitudinal diameters with oblong shapes. Moreover, the intercellular space was increased, and the arrangement was looser than that in the prepigmentation stage (Fig. 1C). As shown in Fig. 1D, there was no color in the unstained jujube pericarp cells, while overall pericarp cells and cell walls displayed orange-yellow and orange-red colors, respectively, after pigmentation. Safranin O-fast green staining results (Fig. 1C) showed that the cell wall prior to pigmentation was blue-green in color with almost no color inside the cell. In contrast, the cells after pigmentation presented a red color, with an increased cell wall thickness. According to the principle of safranin O-fast green staining, safranin displayed the red color of the lignified cell wall, and fast green displayed the blue-green color of cellulose in the cell wall. Our results indicate that the cellulose content in the cell wall of winter jujube was high before pigmentation, while the lignin content was high when the cells became lignified after pigmentation.

As shown in Fig. 1E, the thickness of cuticle cells in the pericarp of winter jujube showed no significant change from the W to R stage, ranging from 9.3 to 10.2 μm. The thickness of the 5–6 layers of epidermal cells decreased with the pigmentation of the pericarp. Specifically, there was no significant change from the W stage (83.6 μm) to the SR-W stage (83.5 μm). However, there was a significant decrease to 59.7 μm at the SR-R stage and 61.2 μm at the R stage, demonstrating a decrease of 20 μm in thickness after pigmentation.

### Observation of pigmentation on the pericarp of winter jujube

**Fig. 2:**
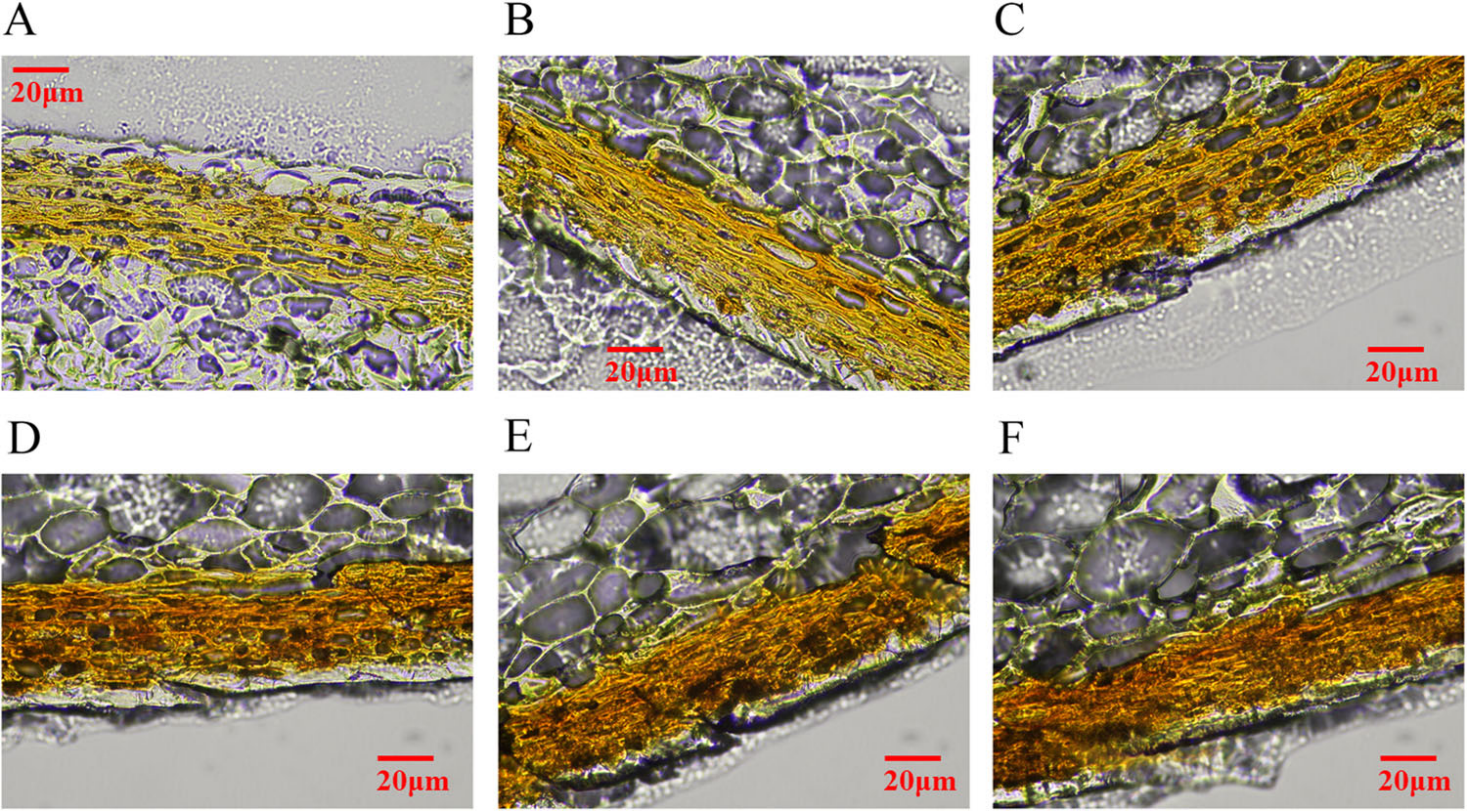
Observation of pigment deposition sites in the pericarp of winter jujube using frozen sections. **A** Frozen section of winter jujube pericarp at the R stage with 5 μm thicknesses. **B** Frozen section of winter jujube pericarp at the R stage with 10 μm thicknesses. **C** Frozen section of winter jujube pericarp at the R stage with 15 μm thicknesses. **D** Frozen section of winter jujube pericarp at the R stage with 20 μm thicknesses. **E** Frozen section of winter jujube pericarp at the R stage with 25 μm thicknesses. **F** Frozen section of winter jujube pericarp at the R stage with 30 μm thicknesses

Figure 2 shows the anatomical structure of frozen sections of winter jujube pericarp at the R stage with six different thicknesses (5, 10, 15, 20, 25, and 30 μm). Consistent with the results from the paraffin sections, the color of the jujube pericarp adhered to the cuticle cells and 5–6 layers of epidermal cells. The epidermal cells of the jujube pericarp were oblong, with a large intercellular space and cellular pigmentation. As the section thickness increased, the color gradually darkened from yellow to orange-red. The sections in Fig. 2A (5 μm) and Fig. 2B (10 μm) were relatively thin and transparent, making it hard to capture the structural details. The sections in Fig. 2E (25 μm) and Fig. 2F (30 μm) were relatively thick, turbid, and inflexible, making it difficult to observe cellular structures. Ultimately, sections at thicknesses of 15 μm (Fig. 2C) and 20 μm (Fig. 2D) illustrated the pericarp cell structure and pigment deposition relatively clearly and vividly. We found through the observations that the epidermal cells of the red jujube pericarp were orange-red in color, with a large amount of pigments deposited on the cell walls.

### Analysis of metabolites related to lignin biosynthesis in winter jujube pericarp during the pigmentation process

**Fig. 3:**
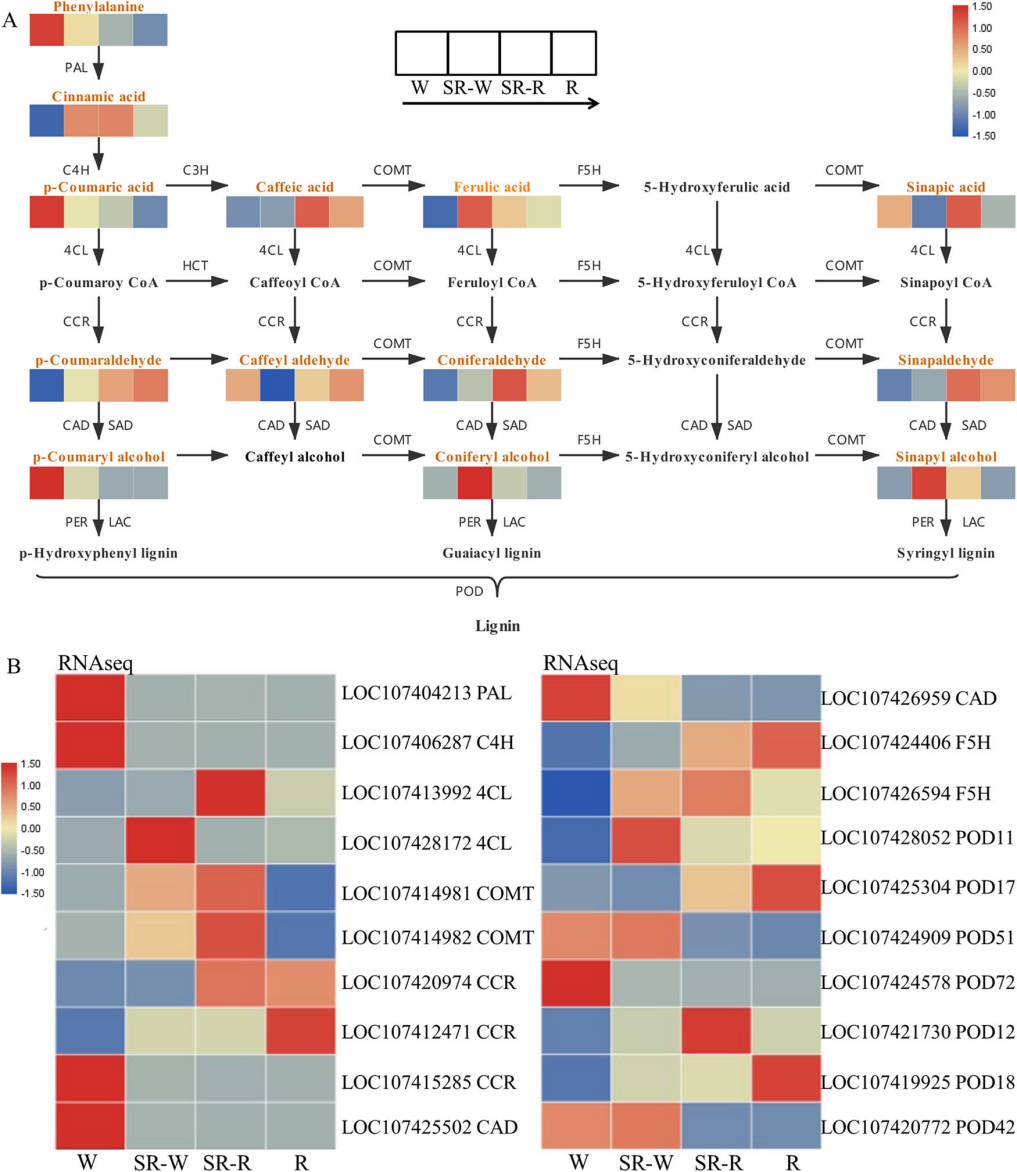
Analyses of metabolites and structural genes related to the lignin biosynthesis pathway during the pigmentation of winter jujube pericarp. **A** Analyses of metabolites involved in lignin biosynthesis during the pigmentation of winter jujube pericarp. Colors from blue to red indicate that the abundance of metabolites is low to high, and a redder color indicates that the abundance of differentially abundant metabolites is high. **B** Heatmap analyses of structural genes involved in lignin biosynthesis during the pigmentation of winter jujube pericarp. Colors from blue to red indicate that the levels of gene expression are low to high, and a redder color indicates that the levels of gene expression are high. W white stage, SR-W white pericarp of the semi-red stage, SR-R red pericarp of the semi-red stage, R: full-red stage

The accumulation of 13 major metabolites related to lignin biosynthesis in winter jujube during the pigmentation process is illustrated in Fig. 3A. Phenylalanine and p-coumaric acid levels were decreased as pigmentation increased. In contrast, p-coumaroyl, sinapyl aldehyde, and coniferyl aldehyde levels were higher in pigmented pericarps at the R and SR-R stages than in unpigmented pericarps at the W and SR-W stages. The contents of coniferyl alcohol and sinapyl alcohol first increased and then decreased, peaking at the SR-W stage. The p-coumaric alcohol content showed a declining trend, and its content was higher at the W stage than at the SR-W stage, with extremely low or no accumulation at the R and SR-R stages. These results indicate that G-S lignin rather than H-lignin is the main monomer of winter jujube pericarp.

The gene expression levels of structural genes (*PAL*, *C4H*, *4CL*, *COMT*, *CCR*, *CAD*, *F5H*, and *POD*) related to lignin biosynthesis in winter jujube were analyzed based on RNA-seq (Fig. 3B). Some genes were selected, and their expression levels were verified by qRT-PCR (Fig. S1). The results showed consistency between the transcriptome and qRT-PCR analysis. Specifically, the expression levels of *PAL* and *C4H* were downregulated during pericarp pigmentation. The expression levels of *COMT* genes (*LOC107414981* and *LOC107414981*) were relatively high at the SR-R stage but low at the R stage. This pattern was different from the accumulating levels of sinapyl aldehyde, coniferyl aldehyde, and lignin. The expression levels of *F5H* (*LOC107424406*) and *CCR* (*LOC107420974*) were relatively high at the SR-R and R stages, consistent with the accumulation of sinapyl aldehyde, coniferyl aldehyde, and lignin. We deduced that *F5H* and *CCR* play key roles in G-S lignin biosynthesis. The expression of *CAD* (*LOC107426959*) was consistent with the accumulation of p-coumeric alcohol and may be the key gene for H-lignin biosynthesis.

### Expression analysis of MYB TFs related to lignin biosynthesis

**Fig. 4:**
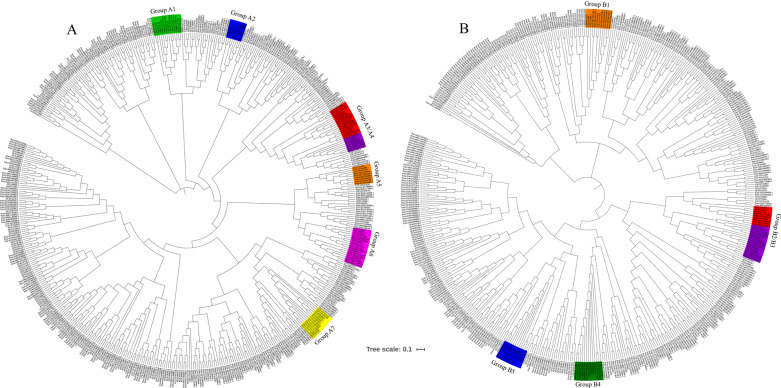
Phylogenetic tree analyses of MYB and NAC TF families in Arabidopsis, poplar, and jujube. **A** Phylogenetic tree of 126 MYB TFs in *Arabidopsis*, 192 MYB TFs in poplar, and 99 MYB TFs in jujube. Accession numbers: AtMYB46 (AT5G12870), AtMYB83 (AT3G08500), AtMYB26 (AT3G13890), AtMYB58 (AT1G16490), AtMYB63 (AT1G79180), AtMYB75 (AT1G56650), AtMYB85 (AT4G22680), AtMYB103 (AT1G63910), PtrMYB3 (Potri.001G267300), PtrMYB20 (Potri.009G061500), PtrMYB6 (Potri.001G005100), and PtrMYB93 (Potri.004G138000.1). **B** Phylogenetic tree of 138 NAC TFs in *Arabidopsis*, 289 NAC TFs in poplar, and 101 NAC TFs in jujube. Accession numbers: AtNST1 (AT2G46770), AtNST2 (AT3G61910), AtNST3 (At1G32770), AtANAC012 (AT1G32770), AtANAC043 (AT2G46770), AtANAC066 (AT3G61910), AtANAC073 (AT4G28500), AtVNI1 (AT5G09330), AtVNI2 (AT5G13180), PtrWND1A (Potri.011G153300), PtrWND2A (Potri.014G104800), PtrWND1B (Potri.001G448400), and PtrWND2B (Potri.002G178700)

A phylogenetic tree was constructed with MYB TFs in jujube, Arabidopsis, and poplar to screen out the potential MYB TFs that regulate lignin biosynthesis in winter jujube. As shown in Fig. 4A, the results showed that the winter jujube MYB TFs *LOC107417581*, *LOC107425254*, and *LOC107418291* were on the same evolutionary branch as poplar *PtrMYB3* and *PtrMYB20*^14^ and Arabidopsis *AtMYB46*^15^, *AtMYB83*^16^, and *AtMYB26*^17^ (Group A3). *LOC107403729* was on a branch with Arabidopsis *AtMYB58* and *AtMYB63*^18^ (Group A2). *LOC107434917* and *LOC107414294* were on a branch with Arabidopsis *AtMYB85*^19^ (Group A1). *LOC107430169* and *LOC107430208* were on a branch with Arabidopsis *AtMYB103*^20^ (Group A4). *LOC107424134*, *LOC107434709*, *LOC107434532*, and *LOC107406469* were on a branch with Arabidopsis *AtMYB75*^19^ (Group A7). *LOC107432692* and *LOC107415078* were on a branch with poplar *PtrMYB6*^21^ (Group A5). *LOC107421590*, *LOC107415776*, and *LOC107404478* were on a branch with poplar *PtrMYB93*^22^ (Group A6).

**Fig. 5:**
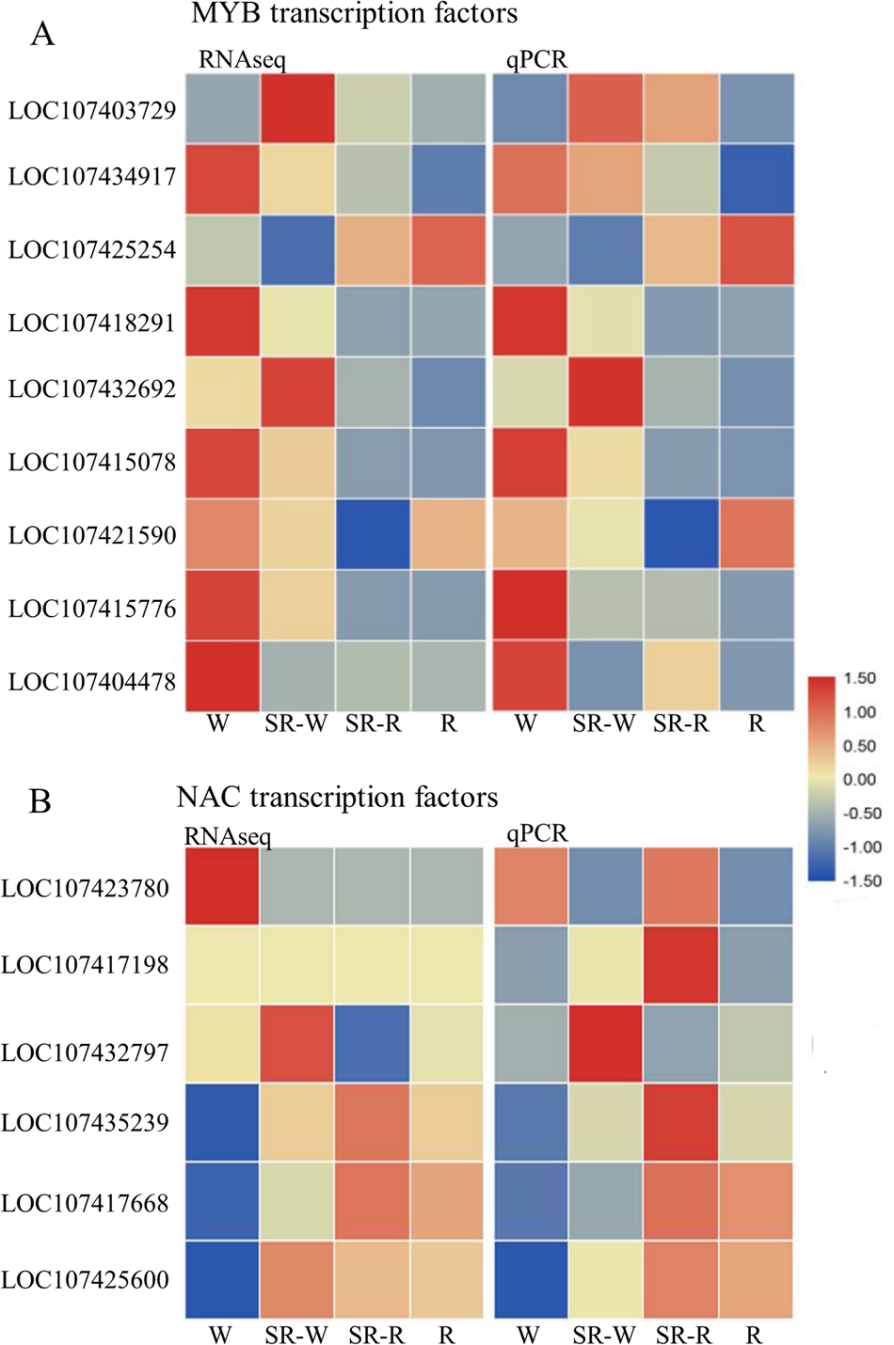
Analyses of the expression levels of MYB and NAC TFs involved in lignin biosynthesis during the pigmentation of winter jujube pericarp. **A** Heatmap displaying the expression levels (RNA-seq and qPCR) of candidate MYB genes involved in lignin biosynthesis during the pigmentation of winter jujube pericarp. **B** Heatmap displaying the expression levels (RNA-seq and qPCR) of candidate NAC genes involved in lignin biosynthesis during the pigmentation of winter jujube pericarp. Colors from blue to red indicate that the levels of gene expression are low to high, and a redder color indicates that the levels of gene expression are high. W white stage, SR-W white pericarp of the semi-red stage, SR-R red pericarp of the semi-red stage, R full-red stage

Seventeen jujube MYB TFs with potential regulation of lignin biosynthesis were screened out by phylogenetic analysis, among which nine TFs showed differential expression levels. The qRT-PCR results showed similar expression trends as the transcriptome analysis (Fig. 5A). In particular, only the expression of *LOC107425254* was in accordance with changes in the lignin content, and the expression levels of *LOC107415078* and *LOC107415776* decreased with increasing lignin content.

### Expression analysis of NAC TFs related to lignin biosynthesis

To screen out the TFs associated with lignin biosynthesis in winter jujube from the NAC family, we used the amino acid sequences of 138 Arabidopsis NAC TFs^23^, 289 poplar NAC TFs (http://planttfdb.cbi.pku.edu.cn), and 101 jujube NAC TFs (http://planttfdb.cbi.pku.edu.cn/) to construct a phylogenetic tree. As shown in Fig. 4B, Arabidopsis *AtNST1*^24^, *AtNST2*, *AtNST3*, *ANAC012*, *ANAC043*, and *ANAC066*^25^ and poplar *PtrWND1A/2A* and *PtrWND1B/2B*^26^ were located on the same branch, which was also shared by the jujube NAC TF *LOC107423780* (Group B2/B3). Arabidopsis *AtANAC073*^25^ was on the same branch as jujube *LOC107417198* and *LOC107432797* (Group B4). Arabidopsis *AtVNI2*^27^ was on the same branch as jujube *LOC107435239* and *LOC107417668* (Group B1). Arabidopsis *AtVNI1*^27^ and jujube *LOC107425600* were on the same branch (Group B5).

Six jujube NAC TFs associated with the regulation of lignin biosynthesis were screened out from the phylogenetic analysis. As shown in Fig. 5B, these six TFs were differentially expressed among samples and showed consistent expression levels between the transcriptome and qRT-PCR analysis, among which *LOC107435239* and *LOC107417668* exhibited relatively higher expression levels after jujube pigmentation, in line with the changes in lignin content.

### Ectopic expression in *Arabidopsis* and pericarp injection in winter jujube

**Fig. 6:**
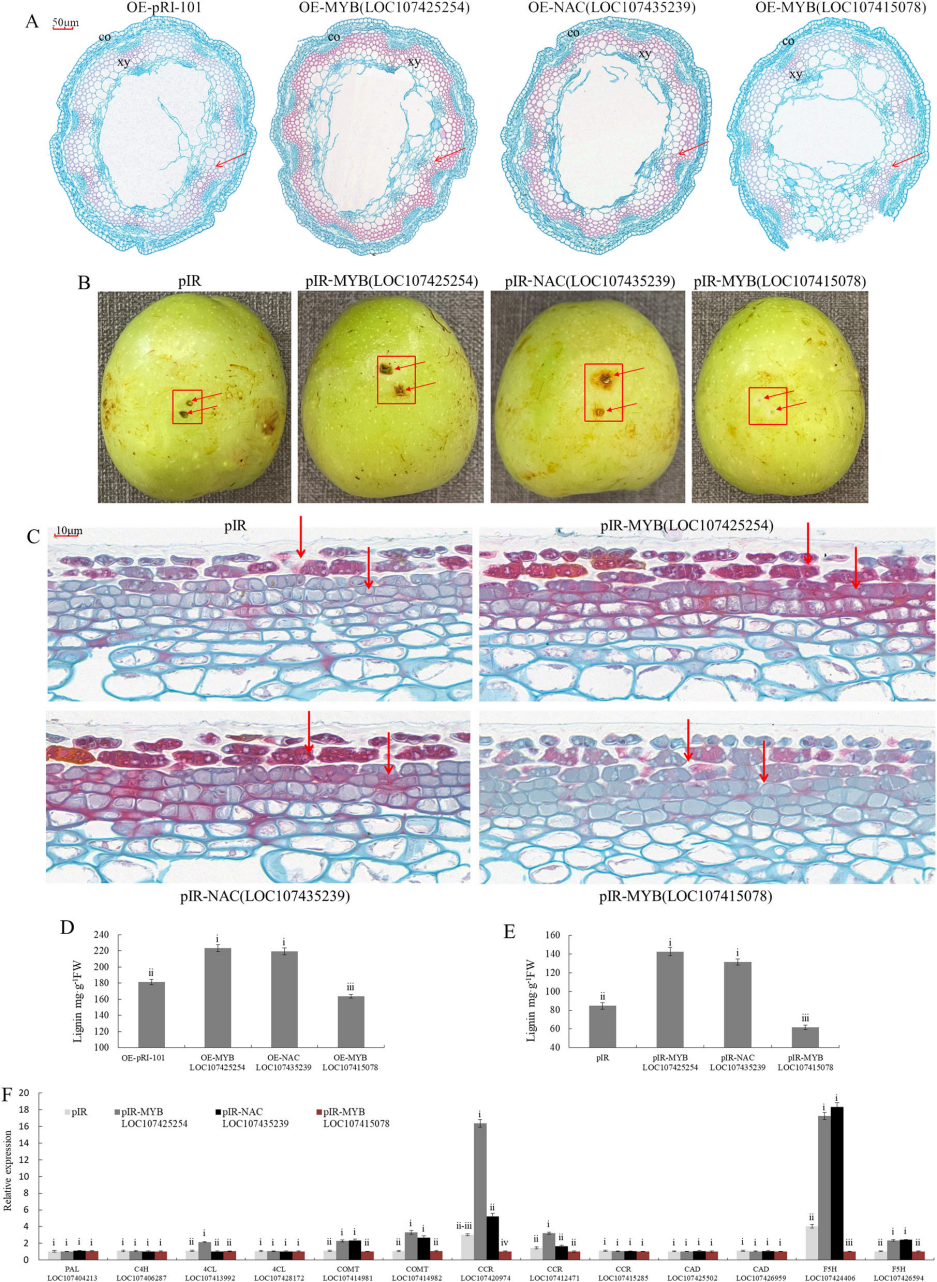
The function of MYB and NAC candidate transcription factors involved in lignin biosynthesis. Significance tests are shown as i, ii, iii, and iv. Different letters above the columns denote significant differences (*P* < 0.01). **A** Cross sections of stems stained with safranin O-fast green in transgenic *Arabidopsis*. OE-pRI-101: overexpressing empty vectors. OE-MYB (*LOC107425254*)/(*LOC107415078*): overexpression of *MYB* (*LOC107425254*) or *MYB* (*LOC107415078*) in *Arabidopsis*, OE-NAC (*LOC107435239*): overexpression of *NAC* (*LOC107435239*) in Arabidopsis. **B** The winter jujube pericarp was injected with the mixed vectors and stored in a phytotron at 25 °C for 3 days. pIR; IL60-1 + IL60-2. pIR-MYB (*LOC107425254*): IL60-1 + MYB (*LOC107425254*)-IL60-2. pIR-MYB (*LOC107415078*): IL60-1 + MYB (*LOC107415078*)-IL60-2. pIR-NAC (*LOC107415078*): IL60-1 + NAC (*LOC107435239*)-IL60-2. **C** The injected winter jujube pericarp stained with safranin O-fast green. **D** Lignin contents of *Arabidopsis* stem. **E** Lignin contents in the winter jujube pericarp around the injection sites. **F** The expression of structural genes associated with lignin biosynthesis in the winter jujube pericarp around the injection sites

The above results identified three MYB (*LOC107425254*, *LOC107415078*, and *LOC107415776*) and two NAC (*LOC107435239* and *LOC107417668*) TFs as candidates associated with lignin biosynthesis. These candidate genes were ectopically expressed in *Arabidopsis* and injected into the winter jujube pericarp. The cross sections of *Arabidopsis* stems stained with safranin O-fast green are shown in Fig. 6A. Overexpression of *MYB* (*LOC107425254*) or *NAC* (*LOC107435239*) in *Arabidopsis* led to deep staining of the stem (Fig. 6A), which was associated with increased lignin levels (Fig. 6D). However, only light staining of stems was observed with *MYB* (*LOC107415078*) overexpression (Fig. 6A), indicating a limited lignin level (Fig. 6D).

The injected winter jujube pericarps are shown in Fig. 6B, and the pericarps stained with safranin O-fast green are shown in Fig. 6C. The overexpression of *MYB* (*LOC107425254*) or *NAC* (*LOC107435239*) caused deep staining in the winter jujube pericarp around the injection sites, while overexpression of *MYB* (*LOC107415078*) resulted in only light staining. In addition, the lignin contents changed with the color of the staining (Fig. 6E). Further gene expression analysis showed that the overexpression of *MYB* (*LOC107425254*) in the winter jujube pericarp could significantly promote the expression of *CCR* (*LOC107420974*) and *F5H* (*LOC107424406*), while overexpression of *NAC* (*LOC107435239*) could only increase the expression of *F5H* (*LOC107424406*) (Fig. 6F). In contrast, the overexpression of *MYB* (*LOC107415078*) in the winter jujube pericarp reduced the expression of *CCR* (*LOC107420974*) and *F5H* (*LOC107424406*) (Fig. 6F).

## Discussion

### Changes in cell structure, cell wall lignification, and pigment deposition during the pigmentation process of winter jujube pericarp

Jujube pericarp pigmentation develops with the ripening process of fruits, which is very complex and involves many physiological processes, including cell senescence, pigment deposition, and metabolite accumulation. Cell senescence includes changes and modifications of cell wall components, as well as aging and death of organs^28^. Similarly, the cell structure of the jujube pericarp also changes with the pigmentation process, which has rarely been reported. Studies on the cell structure of jujube pericarp have mainly focused on the changes in cell structure during fruit cracking and fruit development^29^ rather than ripening and pigmentation. This study revealed that jujube pericarp cells are mainly composed of cuticle cells and epidermal cells (Fig. 1C, D). Moreover, the shape, size, and arrangement of epidermal cells varied with the ripening and pigmentation processes of the fruit. Specifically, the cell shape changed from oblate to oblong, and the arrangement changed from compact to relatively loose during the pigmentation process (Fig. 1C, D), which is consistent with the results of the previous research^4^. Moreover, it has been reported that the epidermal cell thickness and layer number of jujube fruits decrease with fruit ripening. In contrast, our study confirmed the decrease in epidermal cell thickness with fruit ripening, but the layer number remained unchanged (Fig. 1E). This discrepancy between the two studies may be associated with the difference in the jujube varieties used.

The lignification of the jujube pericarp is due to lignin deposition in the cell wall during cell development. Studies have shown organelle degradation, vacuole rupture, and cell content disappearance after cell wall lignification, leading to programmed cell death^30^. Zhao^31^ found that the contents of cellulose and hemicellulose in the pericarp decreased with fruit ripening. Li^32^ showed that the lignin biosynthetic pathway was activated at the transcriptional level during the coloration of green cotton fibers, indicating the function of lignin in pigmentation. In this study, our results showed a decrease in the cellulose content and an increase in the hemicellulose and lignin contents (Fig. 1B). The paraffin sections of jujube pericarp stained with safranin O-fast green revealed the lignification of pigmented epidermal cells reflected by the red color (Fig. 1C). In addition, frozen sections of jujube pericarp at the R stage showed that the epidermal cells were full of chromoplasts, with deposition of a large amount of pigments on the cell wall (Fig. 2). This phenomenon might be caused by the release of pigments (e.g., anthocyanins) from the cell vacuoles during cell wall lignification and cell apoptosis. Furthermore, the orange-red color of thickened cell walls might be associated with the high content of lignin or the polymerization of lignin with the spilled pigments into macromolecular phenols, which needs further verification.

### Analysis of lignin biosynthesis and structural gene expression during the pigmentation process of winter jujube pericarp

It has been shown that the pericarp cells of Chinese jujube change with cell lignification, which is closely related to the accumulation of lignin^33^. Lignin consists of three monomers: H-lignin, G-lignin, and S-lignin. Generally, gymnosperms primarily have G-lignin, monocotyledons primarily have G-S-H lignin, and dicotyledons primarily have G-S lignin^34^. We studied the accumulation of 13 intermediate metabolites of the lignin biosynthesis pathway through metabolomics analysis, and the results showed that p-coumaric alcohol, the precursor of H-lignin, did not accumulate in the pericarp of winter jujube at the SR and R stages. Moreover, coniferyl alcohol, the precursor of G-lignin, and sinapyl alcohol, the precursor of S-lignin, were first increased and then decreased, with a peak at the SR stage (Fig. 3A), indicating the dominance of G-S lignin in the pericarp of winter jujube.

There are eight main structural genes involved in the lignin biosynthesis pathway^35^, among which *PAL*, *C4H*, and *4CL* are the pro-structural genes for lignin biosynthesis. *COMT* and *F5H* are the key genes for G-S lignin biosynthesis. In alfalfa, downregulation of *COMT* expression reduced the content of G-lignin, while S-lignin remained unchanged^36^. Takeda *et al*.^37^ overexpressed *F5H* in rice to increase the S-lignin content. Moreover, overexpression of *BpCCR1* in birch increased lignin content by 14.6%, while knockdown of this gene lowered the lignin content by 6.3%^38^. In our study, the expression levels of *COMT* genes (*LOC107414981* and *LOC107414981*) were relatively high at the SR_R stage but low at the R stage, which was not consistent with the accumulation of coniferyl aldehyde, sinapyl aldehyde, and lignin (Fig. 3 and Fig. S1). The expression levels of *F5H* (*LOC107424406*) and *CCR* (*LOC107420974*) were relatively high at the SR_R and R stages, which was consistent with the accumulation of coniferyl aldehyde, sinapyl aldehyde, and lignin (Fig. 3 and Fig. S1). The results indicated that *F5H* (*LOC107424406*) and *CCR* (*LOC107420974*) might be the key genes for lignin biosynthesis in the pericarp of winter jujube. Notably, we also found that the expression of *CAD* (*LOC107426959*) was consistent with the accumulation of p-coumaric alcohol (Fig. 3), which was in line with a previous report that *CAD* was one of the specific enzymes in the lignin biosynthesis pathway, and the loss of CAD activity impacted lignin composition more than lignin content^39^.

### Regulation of TFs related to lignin during the pigmentation process of winter jujube pericarp

MYB TFs are important regulators of lignin metabolism. Some MYB TFs participate in the regulation of the formation and lignification of secondary cell walls. For example, overexpression of *AtMYB46* and *AtMYB83* activated related structural genes in the lignin biosynthesis pathway, resulting in lignin accumulation and secondary cell wall thickening in *Arabidopsis*^15,16^. In this study, we revealed that the MYB TF *LOC107425254*, which was on the same branch as *AtMYB46* and *AtMYB83* (Fig. 4A), was upregulated with increasing lignin content in the winter jujube pericarp (Fig. 5A), indicating positive regulation of the lignification of winter jujube pericarp cells. Increased lignin content was detected in *Arabidopsis* or winter jujube pericarp overexpressing MYB (LOC107425254) (Fig. 6A–E). Further analysis showed that it could significantly promote the expression of *CCR* (*LOC107420974*) and *F5H* (*LOC107424406*) (Fig. 6F), suggesting that MYB (*LOC107425254*) positively controls lignin content by regulating *CCR* (*LOC107420974*) and *F5H* (*LOC107424406*).

*PtrMYB6* negatively regulated lignin biosynthesis in poplars. Specifically, reduced lignin content and the number of cell layers, as well as thinner cell walls, were observed in the transgenic plants overexpressing *PtrMYB6*^21^. Another study also showed that poplars overexpressing *PtrMYB93* had lower lignin contents. Alternatively, when *PtrMYB93* was knocked out, the expression levels of key structural genes in the lignin biosynthesis pathway were significantly upregulated, and the lignin content was increased^22^. In this study, we found that the levels of the MYB TFs *LOC107415776* and *LOC107415078* were downregulated with increasing lignin content in the winter jujube pericarp (Fig. 5A). Phylogenetic analysis showed that *LOC107415776* and *PtrMYB6* were on the same branch (Fig. 4A), while *LOC107415078* and *PtrMYB93* were on the same branch (Fig. 5A), indicating that they negatively regulate lignin biosynthesis. However, in this study, only MYB (*LOC107415078*) was predicted to negatively regulate lignin biosynthesis by reducing the expression of *CCR* (*LOC107420974*) and *F5H* (*LOC107424406*) (Fig. 6).

NAC TFs, such as AtNST1/2/3^24,25^ and AtVNI1/2^27^ of *Arabidopsis* and PtrWND1A/2A and PtrWND1B/2B of poplar^26^, also play an important role in lignin biosynthesis. In this study, two NAC TFs, *LOC107435239*, and *LOC107417668* were selected for analysis, and their expression levels were upregulated with increasing lignin content (Fig. 5B). Phylogenetic analysis indicated that they were on the same branch as *AtVNI2* (Fig. 4B). *AtVNI2* can inhibit the activation of the *AtVDN7* TF, which specifically regulates the development of secondary cell wall^27^. Therefore, we speculated that *LOC107435239* and *LOC107417668* might be involved in the metabolic process of secondary cell wall development. Only the overexpression of *NAC* (*LOC107435239*) increased lignin content in *Arabidopsis* mutants (Fig. 6A, D). Further analysis also showed that the overexpression of *NAC* (*LOC107435239*) positively regulated lignin biosynthesis in the winter jujube pericarp around the injection sites (Fig. 6B–E) and significantly promoted the expression of *F5H* (*LOC107424406*) (Fig. 6F).

## Conclusion

**Fig. 7:**
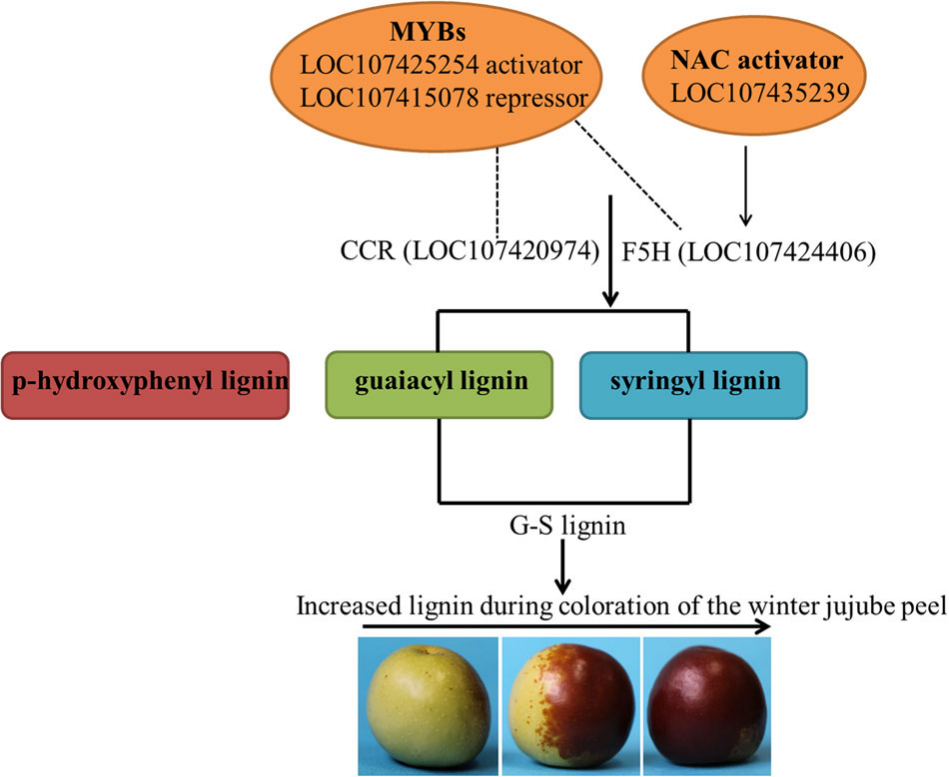
The dotted lines indicate positive and negative regulation, and the arrows indicate positive regulation. The regulatory model of lignin biosynthesis in the pericarp during pigmentation of winter jujube

A regulatory model of lignin biosynthesis in winter jujube pericarp during pigmentation is proposed based on the results from this study (Fig. 7). With the increase in lignin content and the lignification of cell walls during pigmentation of winter jujube pericarp, the thickness of the epidermal cells decreased. The lignin of the winter jujube pericarp was mainly G-S lignin, and *F5H* (*LOC107424406*) and *CCR* (*LOC107420974*) were preliminarily identified as the key genes for G-S lignin biosynthesis. This study also identified an MYB activator (*LOC107425254*) and an MYB repressor (*LOC107415078*) associated with lignin biosynthesis by regulating *CCR* (*LOC107420974*) and *F5H* (*LOC107424406*), while the NAC (*LOC107435239*) TF significantly promoted the expression of *F5H* (*LOC107424406*) and positively regulated lignin biosynthesis. In conclusion, these results revealed the metabolic pathway and key genes controlling lignin biosynthesis during pigmentation of winter jujube pericarp and provide a basis for further research on lignin regulation.

## Materials and methods

### Plant materials

Winter jujube fruits were collected from the germplasm repository of Shandong Institute of Pomology in 2018. The repository was located in the Taishan District of Tai’an City, Shandong Province, China. The growth conditions were a semihumid continental monsoon climate zone, a plain region with clay loam soils, with an average annual temperature of 13 °C, average annual precipitation of 697 mm, and a frost-free period of 195 days. Three 13-year-old healthy jujube plants with similar growth statuses and under unified management were selected for the study. Briefly, ten jujube fruits with homogeneous size, shape, and color were randomly collected from different locations of the sample trees at the white stage (W), semi-red stage (SR), and full-red stage (R). Fruit samples with infection of diseases and pests or mechanical damage were strictly excluded from the study. Different samples were mixed as a biological replicate and used in triplicate for each experiment. After collection, samples were immediately brought back to the laboratory, washed, peeled, frozen in liquid nitrogen, and stored at −80 °C for subsequent uses.

### Determination of insoluble fiber

The lignin content was determined according to the method of Shilton^40^ with slight modification. Briefly, 0.5 g of sample was homogenized with 95% ethanol and centrifuged at 4000 rpm for 10 min. The pellet was washed three times with 95% ethanol and then rinsed three times with ethanol:*n*-hexane (1:1) solution, and the precipitate was collected and dried. The dry mass was placed in the test tube, dissolved in 0.5 mL of 25% bromoacetyl, and incubated at 70 °C in a water bath for 30 min, followed by the addition of 0.9 mL of 2 mol ∙ L^−1^ NaOH to terminate the reaction. Subsequently, 5 mL of glacial acetic acid and 0.1 mL of 7.5 mol ∙ L^−1^ hydroxylamine hydrochloride were added. The mixture was centrifuged for 5 min, 0.1 mL of supernatant was taken and diluted by adding 3.0 mL of glacial acetic acid, and the absorbance value at 280 nm was determined.

The cellulose content was measured by the Cellulase Assay Kit according to the manufacturer’s instructions. Specifically, 0.3 g of sample was first incubated in 80% ethanol for 20 min at 95 °C. After cooling, the sample was centrifuged at 4000 rpm for 10 min. The pellets were vortexed with 1.5 ml of 80% ethanol for 2 min, followed by 2 min of centrifugation at 4000 rpm. After removal of ethanol, the sample was washed with acetone, followed by incubation with 1 mL of reagent I for 15 h. Samples were centrifuged, and the pellets were dried in an oven at 105 °C to constant weights. Five milligrams of dried sample was homogenized using 0.5 mL of deionized water and incubated on ice. Slowly, 0.75 mL of concentrated sulfuric acid was added, and the samples were incubated for another 30 min. The supernatant after centrifugation at 4000 rpm for 10 min was taken and diluted 20 times using deionized water for subsequent measurement. For the preparation of the working solution, 4 mL of reagent III was added into reagent II. Seventy microliters of working solution and 630 μL of concentrated sulfuric acid were added to 300 μL of the prepared sample solution and deionized water, respectively, and incubated at 95 °C for 10 min, and the absorbance at 620 nm was recorded after cooling.

The hemicellulose content was determined by using a commercial hemicellulose detection kit. According to the manufacturer’s instructions, 2 mL of reagent I was added to 0.1 g of the sample, incubated at 90 °C for 10 min, and centrifuged at 5000 rpm for 10 min. The pellets were washed three times with 1 mL of deionized water and kept in an oven at 105 °C until they reached a constant weight, followed by the addition of 1 mL of reagent II. The mixture was incubated at 90 °C for 1 h, followed by the addition of 0.1 mL of reagent III and 1 mL of reagent IV and mixing until the color was reddish. The solution was centrifuged at 5000 rpm for 10 min, and 0.2 mL of the supernatant was thoroughly mixed with 0.15 mL of reagent V and 0.65 mL of deionized water. After 5 min of incubation in a water bath at 90 °C, absorbance at 540 nm was recorded.

### Preparation and observation of paraffin sections

Pericarp samples with a thickness less than 0.2 cm were sliced from the winter jujube fruits from the W stage, SR-W stage, SR-R stage, and R stage cut into 0.3 cm × 0.3 cm pieces, fixed with FAA fixing solution, and incubated in 70% ethanol solution for 12 h. Furthermore, the pericarp samples were dehydrated consecutively in 80% ethanol for 60 min, 90% ethanol for 30 min, and 100% ethanol for 15 min. The dehydrated samples were further incubated sequentially in anhydrous ethanol/xylene solution for 15 min, in xylene solution for 3 min, and in paraffin/xylene for 30 min, followed by paraffin embedding for 80 min. The embedded samples were sliced into sections with a thickness of 5 μm. The paraffin slices were picked out with toothpicks and placed on slides, developed with warm water at 35 °C, placed on a slide coated with a thin layer of protein glycerin, and dried in an oven at 38 °C.

The samples at the same stage were then treated with safranin O-fast green staining as follows: the sections were placed in xylene I for 20 min, xylene II for 20 min, anhydrous ethanol I for 5 min, anhydrous ethanol II for 5 min, and 75% ethanol for 5 min, and then rinsed with ultrapure water. The treated samples were stained in 0.1% safranin solution for 2 h, rinsed with ultrapure water, and placed in 50, 70, and 80% ethanol for 5 s. The samples were then stained with 0.15% solid green dye for 60 s, rinsed with ultrapure water and glacial acetic acid solution, dehydrated with 95% ethanol for 10 s, dehydrated with anhydrous ethanol for 10 s, processed in xylene for 5 min, and sealed with neutral glue. Nonstained and stained slices were observed under an optical microscope (Olympus BX53) and photographed. Changes in the epidermal cell layer and cell structure of the jujube pericarp were observed. The thicknesses of cuticle and epidermal cells were measured with CaseViewer software (The Digital Pathology Company).

### Preparation and observation of frozen sections

Fresh winter jujube fruits at the R stage were cut into 0.5 cm × 0.5 cm × 0.2 cm pieces, placed in the sample disc, and surrounded by OCT embedding drops. The sample disc was slowly placed into the liquid nitrogen tank. When the disc bottom reached the liquid nitrogen, the disc was held for 10–20 s before being immersed in liquid nitrogen. The frozen samples were sliced into sections with thicknesses of 5, 10, 15, 20, 25, and 30 μm. The sections were observed and photographed under an optical microscope (Olympus BX53). The number of epidermal cell layers, the change in cell structures, and pigment deposition were recorded.

### Ultraperformance liquid chromatography-mass spectrometry (UPLC-MS) analysis

Approximately 0.1 g of winter jujube pericarp sample was ground into powder in liquid nitrogen, and 500 μL of 80% methanol solution containing 0.1% formic acid was added; then, the sample was vortexed and placed in an ice bath for 5 min. After centrifugation at 15,000 rpm for 10 min at 4 °C, the sample was diluted with a 53% methanol solution and centrifuged at 15,000rpm for 20 min at 4 °C. The supernatant was used for UPLC-MS analysis. Aliquots from each test sample were mixed as quality control (QC) samples.

The UPLC assay was performed on a Shim-pack Shimadzu CBM30A equipped with a Waters Acquity UPLC HSS T3 C18 column of 1.8 µm (2.1 mm × 100 mm). The experimental conditions were as follows. flow rate: 0.4 ml min^−1^; column temperature: 40 °C; injection volume: 2 μL; mobile phase A: 0.04% acetic acid aqueous solution, mobile phase B: 0.04% acetonitrile solution. Sample elution was established by mixing mobile phase A/mobile phase B (V/V) in a linear gradient: 0 min was 95:5, 11.0 min was 5:95, 12.0 min was 5:95, 12.1 min was 95:5, and 15.0 min was 95:5.

Mass spectrum analysis was carried out by a triple four-pole tandem mass spectrometer (Applied Biosystems 6500 QTRAP) with the following parameters: temperature 500 °C, voltage 5500 V, curtain gas (CUR) 25 psi, and collision-activated dissociation (CAD) as high.

### Qualitative and quantitative analysis of metabolites and data analysis

Based on the Metware Database (MWDB) and other public metabolite information databases, metabolite characterization was carried out according to the secondary spectral information. During the analysis, isotope signals, repeated K^+^, Na^+^, and NH4^+^ ion signals, and repetitive signals of fragment ions contained in other larger molecules were removed. Metabolites were quantitatively analyzed by multiple reaction monitoring (MRM) using Triple Quad mass spectrometry.

The metabolite data were analyzed using Analyst 1.6.1 software (AB Sciex, Ontario, Canada). Principal component analysis (PCA) was used to analyze the differences in population metabolism among samples and the degree of variation among samples within a group. The metabolites were analyzed by partial least square discriminant analysis (OPLS-DA), and the metabolites with variable importance in projection (VIP) ≥1 and *P* value ≤0.5 were identified as differentially abundant metabolites. Hierarchical clustering analysis (HCA) and metabolite correlation analysis were used to reveal the relationship between metabolites and samples.

### Transcriptome analysis

Total RNA was extracted from the pericarp of winter jujube using an RNAprep Pure Polysaccharide polyphenol total plant RNA extraction kit (TransGen Biotech, Beijing, China). The qualified total RNA was used for subsequent library construction and sequencing by the Illumina NovaSeq 6000 platform.

The reference genome sequence and functional gene annotation files were retrieved from https://ftp.ncbi.nlm.nih.gov/genomes/all/GCF/000/826/755/GCF_000826755.1_ZizJuj_1.1. Hisat2 V2.0.5 was used to compare clean paired-end reads with the reference genome. Reads of each gene in each sample were calculated using featureCounts V1.5.0-p3, and the value of fragments per kilobase of exon per million mapped reads (FPKM) of each gene was calculated based on the length of the gene.

DESeq2 R software (version 1.16.1) was used to compare expression differences between paired groups. Genes with *P* < 0.05 and | log2(fold change) | >0 after correction were considered significantly differentially expressed genes for subsequent analysis.

Gene Ontology (GO) enrichment and KEGG enrichment analysis of differentially expressed genes were achieved by clusterProfiler R software, in which the gene length bias was corrected. GO terms and KEGG terms with adjusted *P* < 0.05 were used as significantly enriched functional terms for subsequent analysis.

### Quantitative real-time polymerase chain reaction (qRT-PCR)

The selected genes and TFs were verified by qRT-PCR. Specific primers were designed using Primer 6.0 according to the whole genome sequences of winter jujube. The TransStart^®^ Top Green qPCR SuperMix kit was used for detection. Each sample was analyzed in triplicate, with *ZjActin* used as the internal reference gene. The Ct values were read under default conditions, and the 2^−ΔΔCT^ method was used for data analysis^41^.

### Ectopic expression in *Arabidopsis* and pericarp injection of winter jujube

Ectopic expression was carried out as described by Wang et al.^42^, with slight alterations. The recombinant plasmids containing candidate genes were transformed into *Agrobacterium tumefaciens* GV3101, which was utilized to infect *Arabidopsis*. The T1 transgenic Arabidopsis plants were then selected by plating onto MS medium containing hygromycin. The hygromycin-resistant seedlings were moved to soil and grown in a growth chamber (Ningbo-Jiangnan, http://www.nbjnyq.com/). The T2 seeds were then collected and grown as described above, and the lignin contents of their stems were determined.

Pericarp injection assays were carried out as described previously^43^. The overexpression of viral vectors MYB (*LOC107425254*)-IL60–2, MYB (*LOC107415078*)-IL60-2, and NAC (*LOC107435239*)-IL60-2 was generated by inserting the corresponding CDSs into the IL60-2 vector. The IL60-1 vector was used as an auxiliary plasmid. The mixed vectors (IL60-1: IL60-2 = 1: 1; 500 μL: 100 μM acetosyringone, 10 mM 2-morpholinoethanesulfonic acid) were injected into the fruit pericarp using a 1 mL medical syringe. Then, the fruits were stored in a phytotron at 25 °C for 3 days.

### Statistical analysis

All results shown represent the mean of three independent assays. Significant differences between groups were determined by Duncan’s new multiple range test, with significance tests denoted as i, ii, iii, and iv. Different lowercase letters in figures and tables denote significant differences (*P* < 0.05).

## Supplementary information

Supplemental Figure 2

Supplemental Figure 1

## Data Availability

Supporting data not included in the manuscript are stored in (https://pan.baidu.com/share/init?surl=G_N60yxSOw-b4zKyHsNMgg, password: 2021).
