# Supplementary figures and images for: The regulation of cell wall lignification and lignin biosynthesis during pigmentation of winter jujube
Source: Hortic Res. 2021 Nov 1;8:238. doi: 10.1038/s41438-021-00670-4 (PMC8558337; doi:10.1038/s41438-021-00670-4)

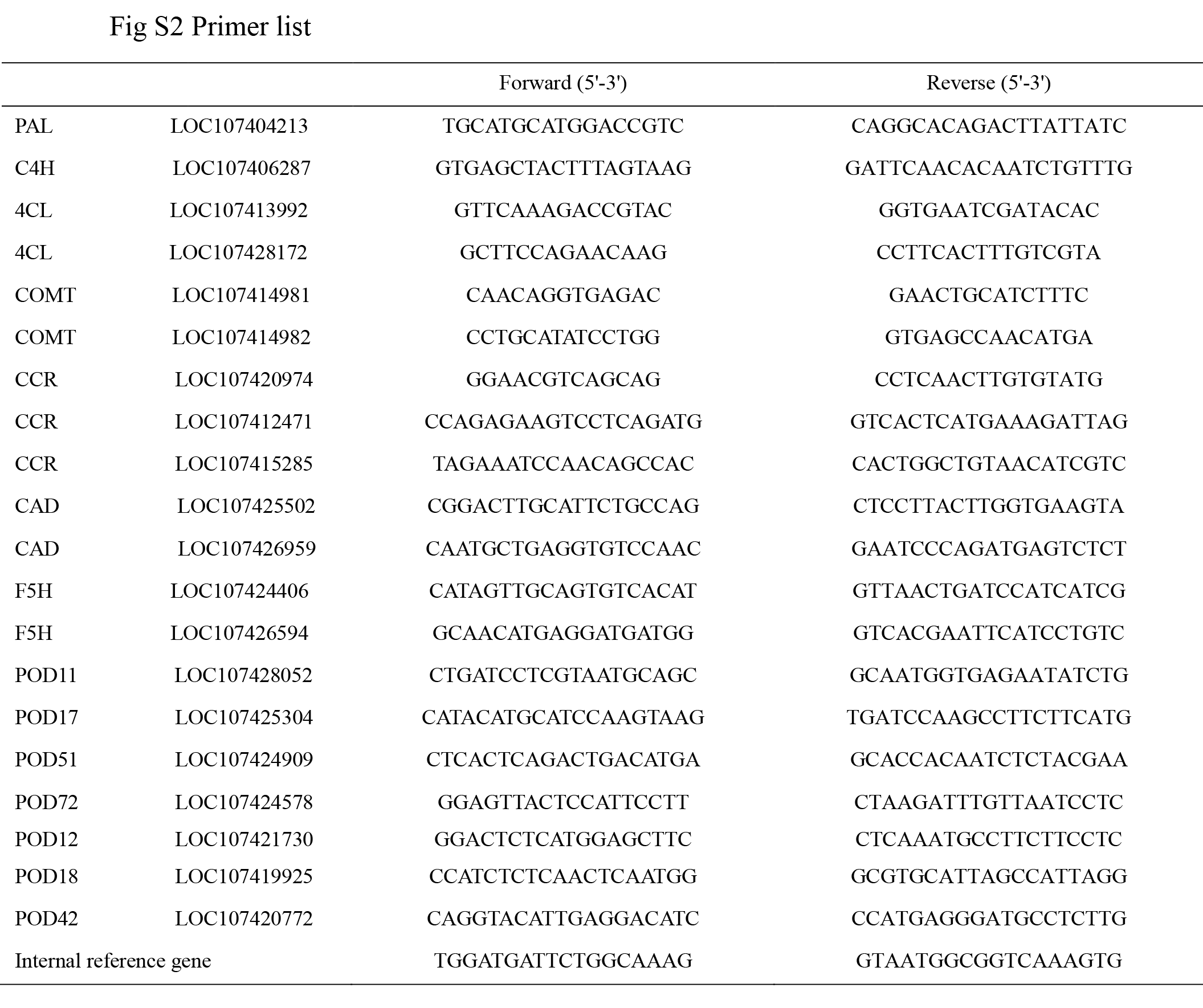

Supplement: Supplementary file 1 — Supplemental Figure 2 [file 41438_2021_670_MOESM1_ESM.tif]

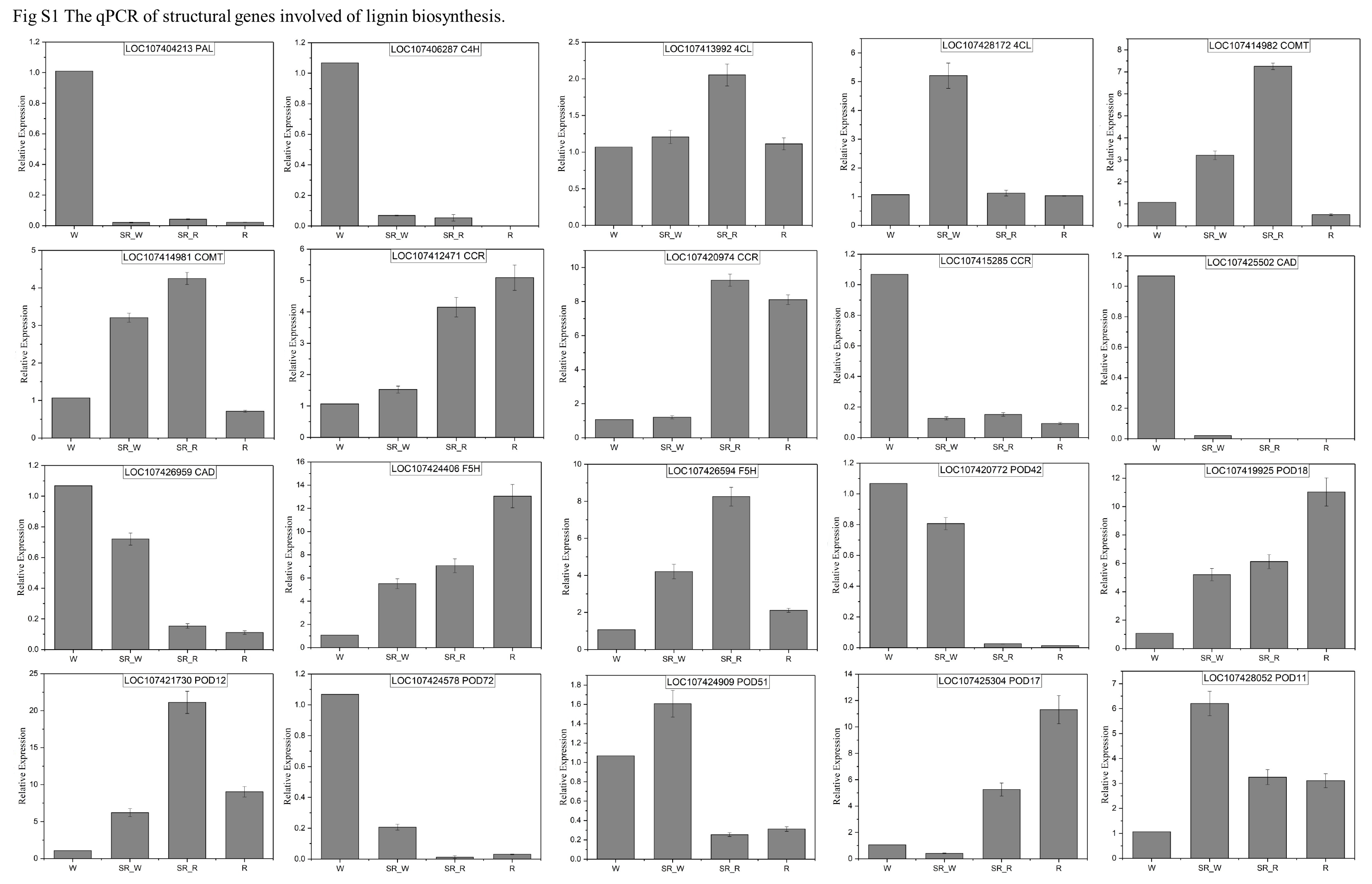

Supplement: Supplementary file 2 — Supplemental Figure 1 [file 41438_2021_670_MOESM2_ESM.tif]
